# Zinner syndrome in pediatric patients: rare disease leading to challenging management

**DOI:** 10.3389/fped.2024.1353960

**Published:** 2024-01-24

**Authors:** Ottavio Adorisio, Cinzia Orazi, Lorenzo Maria Gregori, Francesco De Peppo, Massimiliano Silveri

**Affiliations:** ^1^ Pediatric Surgery Unit, Bambino Gesù Children’s Hospital, IRCCS, Rome, Italy; ^2^ Diagnostic Imaging Unit, Bambino Gesù Children’s Hospital, IRCCS, Rome, Italy; ^3^ Pediatric Andrological and Gynecological Surgery Unit, Bambino Gesù Children’s Hospital, IRCCS, Rome, Italy

**Keywords:** Zinner syndrome, seminal vesicle, pelvic pain, renal agenesis, ejaculatory duct

## Abstract

**Introduction:**

Zinner syndrome (ZS) is the association of seminal vesicle cysts, ipsilateral ejaculatory duct obstruction, and ipsilateral renal agenesis. This condition is very rare in children and both diagnosis and treatment may be challenging. We reviewed the clinical presentation and treatment describing our experience with a series of three patients.

**Methods:**

From January 2016 to January 2021, three patients (patients 1, 2, and 3) with symptomatic ZS, aged 2, 15, and 17 years, respectively, were diagnosed and treated. All three patients were symptomatic, manifesting pelvic pain and dysuria. The diagnosis was made by physical examination, ultrasonography, and abdominopelvic MRI. Patient 1 underwent open surgery, while for patients 2 and 3, laparoscopic excision was performed.

**Results:**

The renal agenesis regarded the left side in patients 1 and 3, and the right side in patient 2. In all cases, the cystic complex was excised. The mean operating time was 4 h and the mean hospitalization time was 5 days (range 4–6 days). The mean follow-up period was 5 years (range 2–5 years). Patients 1 and 3 showed a complete resolution of the symptoms during postoperative follow-up. In patient 2, clinical symptoms relapsed because of the persistence of a 9 mm cyst requiring a redo laparoscopic excision.

**Conclusions:**

Seminal vesicle cyst with ipsilateral renal agenesis, even if rare in pediatric age, should be suspected in young male patients presenting with pelvic cystic masses, pelvic pain, dysuria, and ipsilateral renal absence. Conservative management should be reversed to asymptomatic patients. Surgical treatment is mandatory in symptomatic cases and the preferred approach is minimally invasive surgery to magnify the operating field to spare anatomical structures, primarily the contralateral vas deferens. Radicality is crucial to avoid the persistence of symptoms and the need for reintervention.

## Introduction

Zinner syndrome (ZS) is the association of seminal vesicle cysts (SVCs), ipsilateral ejaculatory duct obstruction, and ipsilateral renal agenesis. First described by Zinner in 1914, it is a very rare disease (1). This syndrome is a result of the abnormal development of the ureteric bud in early phases of embryogenesis leading to ipsilateral anomalies of both the kidney and other mesonephric duct-derived structures. The majority of patients with ZS are diagnosed in the second to fourth decades of life when they become symptomatic, showing bladder irritation, urinary obstruction, perineal pain, and infertility (2). However, ZS in prepubertal patients is generally asymptomatic and often detected incidentally (2, 3). There are only a few reports of pediatric patients in the literature, especially in infancy (2, 3). The widespread use of ultrasound (US) during pregnancy and infancy allowed us to find an increasing number of cases of unilateral renal agenesis or multicystic kidney disease (MCDK) (4). The presence of these anomalies should alert the physicians for other ipsilateral or contralateral genitourinary malformations, such as SVCs in male patients. Diagnosis and treatment of ZS may be very challenging, due to its rarity. We reviewed our experience with the clinical presentation and treatment of three pediatric patients.

### Case 1

**Figure 1:**
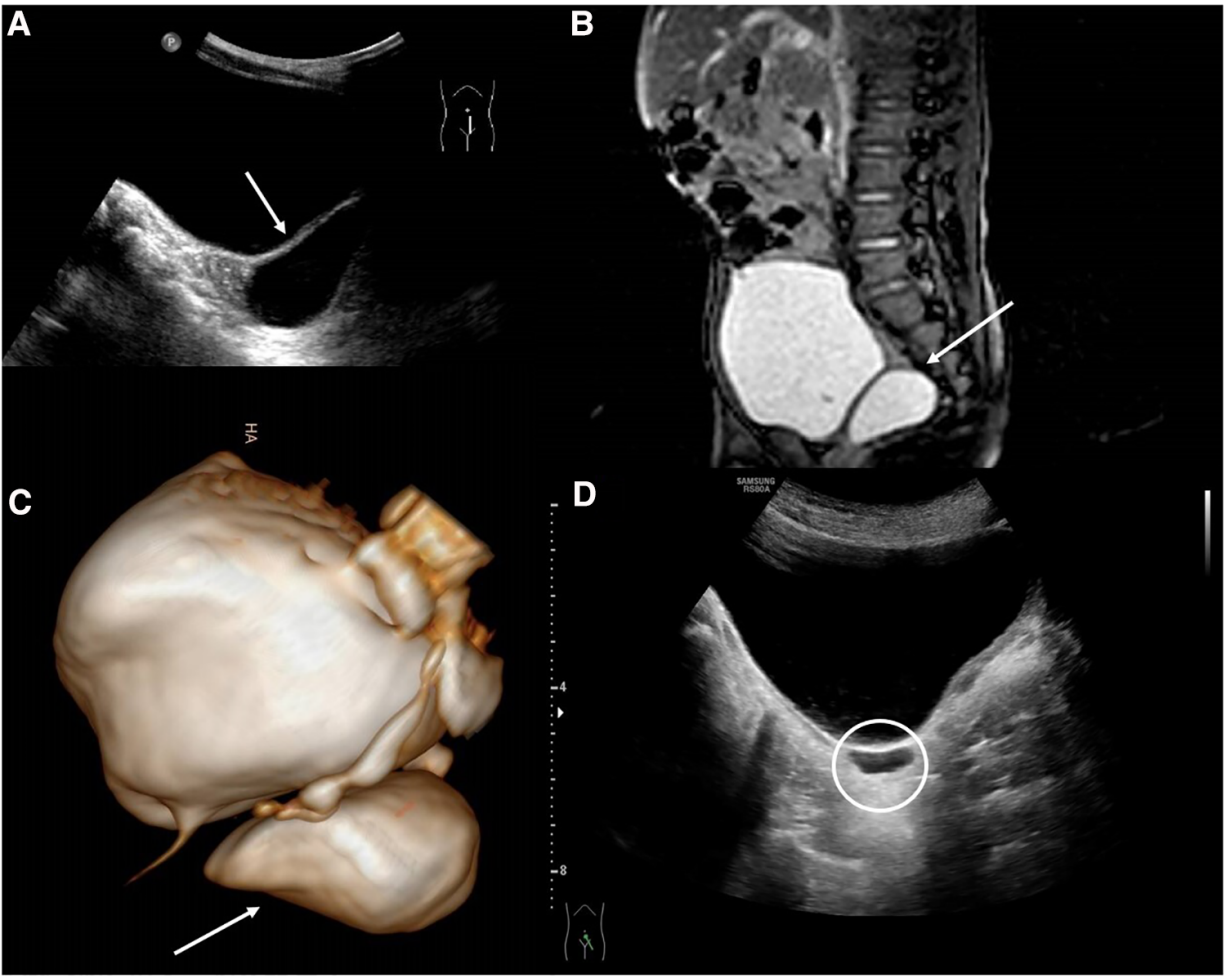
(**A**) US study showed a 36 mm × 16 mm right retrovesical fluid-filled cyst; (**B**) MRI showing the retrovesical SVC; (**C**) 3D MRI reconstruction; (**D**) postoperative US showing; the persistence of the 4 mm × 14 mm cyst.

A 2-year-old male patient, with an antenatal diagnosis of left renal agenesis, came to our Emergency Department (ED) for a history of one-month lower abdominal pain and dysuria. A US study showed a 36 mm × 16 mm right retrovesical fluid-filled cyst (Figure 1). The magnetic resonance imaging (MRI) confirmed the absence of the right kidney, with a well-defined, retrovesical cystic lesion (Figure 1). A cystoscopy was performed. The right ureteral orifice was absent and only the right ureteric orifice was detected. Surgical removal was carried out. Through a Pfannenstiel incision, using loupes magnifying glasses, the cyst was carefully excised, trying to avoid injuries to the bladder, blood vessels, and nerve bundles. The postoperative period was uneventful and the baby was discharged on the fifth postoperative day. After a 7-year follow-up, serial US studies showed the persistence of a 4 mm × 14 mm cyst (Figure 1), in the absence of both abdominal and urinary symptoms.

### Case #2

**Figure 2:**
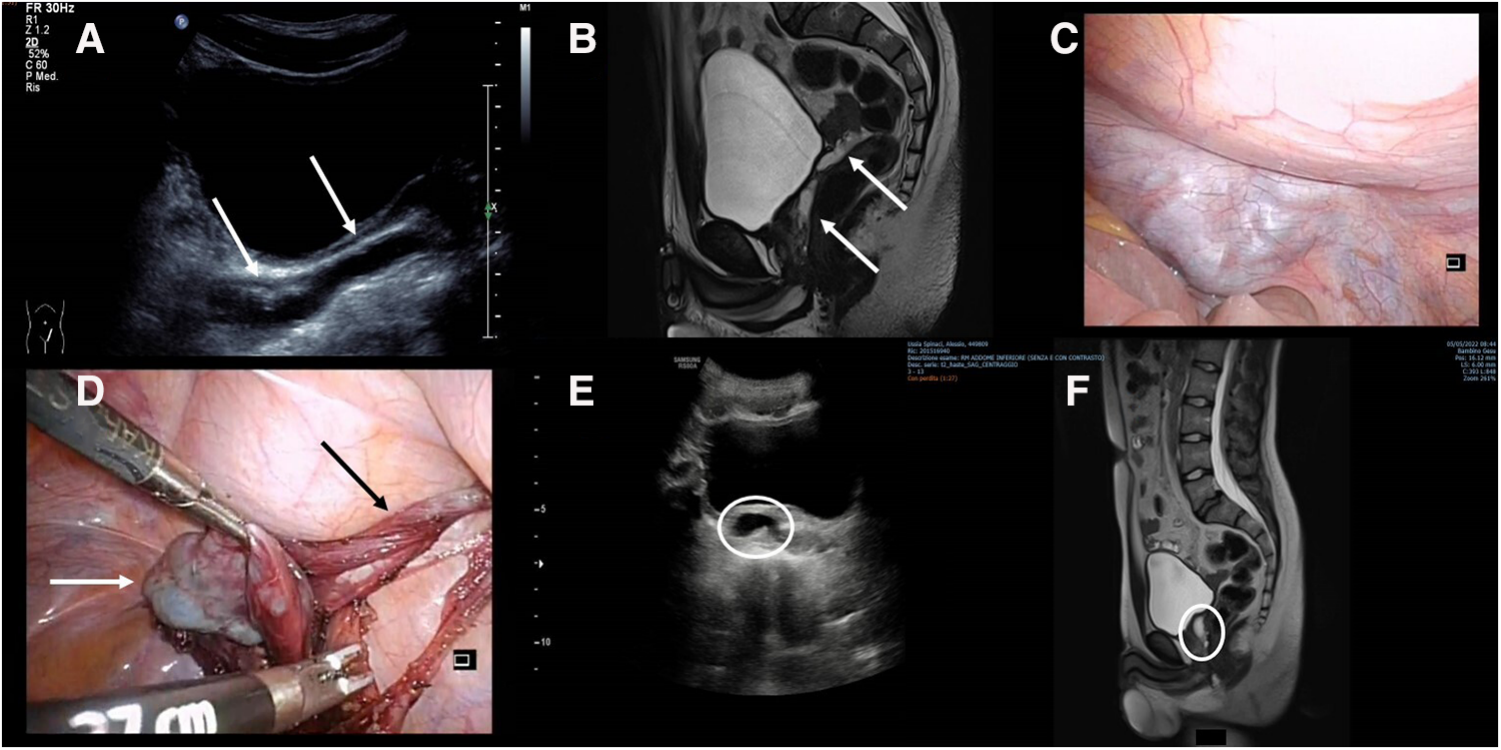
(**A**) US showing the presence of a 10 mm × 50 mm unilocular dilatation of the seminal vesicle (white arrows); (**B**) MRI confirming the presence of the retrovesical cyst (white arrows); (**C**) laparoscopic view of the cystic lesion; (**D**) intraoperative view of the SVC (white arrow) and vas deferens (black arrow); (**E**) postoperative US showing the residual SVC; (**F**) MRI showing the sagittal view of the residual SVC.

A 15-year-old male patient came to our attention for pelvic–perineal pain and stranguria. He had a prenatal diagnosis of right MCDK. Serial US of the abdomen showed the disappearance of the right kidney at the age of 8 years. The US performed in the ED confirmed the absence of the right kidney with the presence of a 10 mm × 50 mm unilocular dilatation of the ipsilateral seminal vesical (Figure 2). An MRI confirmed the complete absence of the right kidney, with well-defined, cystic, mass-like lesions (Figure 2). Because the patient was symptomatic, laparoscopic excision of the seminal vesical dilatation cyst was carried out (Figure 2). Due to the high risk of both nerve bundles and contralateral SVC/vas deferens injuries, before the procedure the patient was asked to perform the cryopreservation of the semen. The postoperative period was uneventful and the patient was discharged on the fourth postoperative day. Six months after the procedure the patient was admitted to our division for the onset of perineal pain. The US and the MRI of the pelvis showed the presence of 14 mm residual SVCs (Figure 2). Due to the persistence and the worsening of the symptoms, a second laparoscopic look was undertaken with the excision of the residual cyst. The postoperative course was uneventful, and the patient was discharged on the fifth postoperative day. Two years after the procedure, the patient is in good condition with a total absence of symptoms.

### Case 3

**Figure 3:**
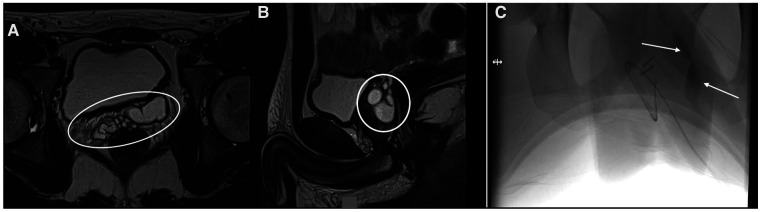
(**A,B**) MRI showing the SVC (white arrows); (**C**) genitography performed using a 3Ch ureteral catheter inserted into the left ejaculatory duct contrasting the left SVC.

A 19-year-old male patient came to our attention for a history of 9 years of pelvic–perineal pain and stranguria with several admissions to the ED. Left renal agenesis was detected during pregnancy. The US of the abdomen performed at every admittance to the ED confirmed the absence of the left kidney but no abdominal or pelvic cystic lesions were detected. An MRI of the abdomen and pelvis was carried out showing a multi-lobulated mass measuring 17 mm × 62 mm with bilateral extension of the SVCs (Figure 3). A cystoscopy was performed. The left ureteral orifice was absent and only the right ureteric orifice was detected in its orthotopic position. An orifice was identified at the bottom of the prostatic urethra and proximally to the verumontanum, and it looked partially closed by a membrane. It opened into a dilated seminal vesicle. A 3Ch ureteral catheter was inserted into this orifice to perform a vesiculography (Figure 3C). Due to the high risk of nerve bundles and contralateral SVC/vas deferens injuries, before the procedure this patient, too, was asked to perform the cryopreservation of the semen. Laparoscopic excision was then carried out. The postoperative period was uneventful and the patient was discharged on the fourth postoperative day. There was no postoperative complication and the patient’s symptoms improved immediately. After 12 months follow-up, the patient remains completely asymptomatic.

## Discussion

The Zinner syndrome is an uncommon disease characterized by the association of SVCs, ipsilateral ejaculatory duct obstruction, and kidney agenesis or MCKD (4). Congenital SVCs are more common than secondary cysts and are often associated with other congenital genitourinary anomalies, especially ipsilateral renal agenesis (67%–75%), because of their common embryologic origin (5). An incomplete migration of the ureteric bud, arising from the proximal portion of the mesonephric duct, fails to join the metanephros, resulting in agenesis or dysplastic kidney (6, 7). This may cause poor drainage of the seminal vesicles fluid, resulting in a cystic formation. The almost total absence of symptoms in the first years of life, its rarity, and the wide spectrum of potentially confusing imaging findings, combined with the lack of familiarity of the physicians, make this syndrome difficult to diagnose. The age of presentation varies from 1 to 68 years (8). In a great part of cases, symptoms begin with the onset of sexual life due to the enlargement of the cyst from seminal fluid accumulation. The real incidence of ZS in pediatric patients is very difficult to define (5). Sheih et al. found 13 cystic dilatations among 280,000 infants and children screened with the US affected by ipsilateral renal anomalies (0.00214%) (5). Conversely, unilateral renal agenesis or MCDK are frequently observed in newborns and infants, especially in the last years due to the increasing use of prenatal and postnatal US studies (9). These anomalies should alert for other ipsilateral genitourinary anomalies. In the case of cystic anomalies of the pelvis, there are some important differential diagnoses to rule out such as prostatic utricle cysts (PUCs), given their central position and communication with the urethra. However, these anomalies are often associated with Veisco-Ureteral Reflux (VUR), hypospadias, and/or undescended testes. Another differential that should be considered is the Müllerian duct cysts (MDCs). Usually, MDCs do not communicate with the urethra (10). Conversely, patients with MDCs often have normal external genitalia with the pelvic cyst in the midline, as seen in ZS. Common symptoms at presentation are dysuria (37%), perineal pain (29%), and epididymitis (27%) (11, 12). Rarely, the cysts can be greater than 12 cm, leading to obstructive symptoms of the bladder and rectum (13). Smaller cysts, less than 5 cm in size, may present with vague, varied symptoms, such as ejaculatory pain, hematospermia, and infertility. Patients affected by ZS with smaller cysts (less than 2 cm) may remain asymptomatic and the presence of the cysts becomes evident during instrumental examinations performed for other reasons (14). Rare reports of papillary adenocarcinoma and squamous cell carcinoma have been reported in SVCs associated with renal agenesis between 17 and 41 years of age, and no patient in the pediatric age group developing seminal vesicle malignancy has been mentioned in the literature (15, 16).

Treatment of ZS remains a debated topic and the definition of the gold standard is still controversial. Conservative management of SVCs is commonly accepted for asymptomatic or poorly symptomatic patients, with prolonged monitoring (2). In the literature, more than 80% of pediatric cases showed no clinical symptoms during a long-term follow-up, but they should stay under extended long-term follow-up (2, 3). Surgical intervention, the treatment of choice in the adult population, should be considered also in children and adolescents with symptomatic disease (17).

## Conclusions

The minimally invasive approach (laparoscopic/robotic), in symptomatic pediatric patients, should be preferred due to the excellent magnification of the anatomical structures, reducing both the risk of nerve bundle injuries and hospital stay (18). Radicality is crucial to avoid the persistence of symptoms and the need for reoperation.

## Data Availability

The original contributions presented in the study are included in the article/Supplementary Material, further inquiries can be directed to the corresponding author.

## References

[1] Kavanaki A Vidal I Merlini L Hanquinet S. Congenital seminal vesicle cyst and ipsilateral renal agenesis (Zinner syndrome): a rare association and its evolution from early childhood to adolescence. Eur J Pediatr Surg Rep. (2015) 3:98–102. 10.1055/s-0035-1555605 PMC4712056 26788458

[2] Cascini V Di Renzo D Guerriero V Lauriti G Lelli Chiesa P. Zinner syndrome in pediatric age: issues in the diagnosis and treatment of a rare malformation complex. Front Pediatr. (2019) 9(7):129–35. 10.3389/fped.2019.00129 PMC6465625 31024871

[3] Rose MOR Ravi Banthia JMO Tamboli Z Lal H. Zinner syndrome: a rare diagnosis in infancy. BMJ Case Rep. (2022) 15(5):e248558. 10.1136/bcr-2021-248558 35589266 PMC9121408

[4] Schukfeh N Kuebler JF Schirg E Petersen C Ure BM Glüer S. Dysplastic kidney and not renal agenesis is the commonly associated anomaly in infants with seminal vesicle cyst. BJU Int. (2009) 103:816–9. 10.1111/j.1464-410X.2008.08072.x 19040535

[5] Sheih CP Hung CS Wei CF Lin CY. Cystic dilatations within the pelvis in patients with ipsilateral renal agenesis or dysplasia. J Urol. (1990) 144:324–7. 10.1016/S0022-5347(17)39444-2 2197430

[6] Chen HW Huang SC Li YW Chen SJ Sheih CP. Magnetic resonance imaging of seminal vesicle cyst associated with ipsilateral urinary anomalies. J Formos Med Assoc. (2006) 105:125–31. 10.1016/S0929-6646(09)60333-8 16477332

[7] van den Ouden D Blom JH Bangma C de Spegeller AH. Diagnosis and management of seminal vesicle cysts associated with ipsilateral renal agenesis: a pooled analysis of 52 cases. Eur Urol. (1998) 33:433–40. 10.1159/000019632 9643661

[8] Terai A Tsuji Y Terachi T Yoshida O. Ectopic ureter opening into the seminal vesicle in an infant: a case report and review of the Japanese literature. Int J Urol. (1995) 2:128–31. 10.1111/j.1442-2042.1995.tb00439.x 7553286

[9] Merrot T Lumenta DB Tercier S Morisson-Lacombes G Guys JM Alessandrini P. Multicystic dysplastic kidney with ipsilateral abnormalities of genitourinary tract: experience in children. Urology. (2006) 67:603–7. 10.1016/j.urology.2005.09.062 16527586

[10] Fiaschetti V Greco L Giuricin V De Vivo D Di Caprera E Di Trapano R Zinner syndrome diagnosed by magnetic resonance imaging and computed tomography: role of imaging to identify and evaluate the uncommon variation in development of the male genital tract. Radiol Case Rep. (2017) 12(1):54e58. 10.1016/j.radcr.2016.10.007 28228879 PMC5310246

[11] King BF Hattery RR Lieber MM Berquist TH Williamson B Jr Hatman JW. Congenital cystic disease of the seminal vesicle. Radiology. (1991) 178:207–11. 10.1148/radiology.178.1.1984306 1984306

[12] Rappe BJ Meuleman EJ Debruyne FM. Seminal vesicle cyst with ipsilateral renal agenesis. Urol Int. (1993) 50:54–6. 10.1159/000282451 8434428

[13] Heaney JA Pfister RC Meares EM. Giant cyst of the seminal vesicle with renal agenesis. AJR Am J Roentgenol. (1987) 149:139–40. 10.2214/ajr.149.1.139 3495973

[14] Kenney PJ Leeson MD. Congenital anomalies of the seminal vesicles: spectrum of computed tomographic findings. Radiology. (1983) 149:247–51. 10.1148/radiology.149.1.6611933 6611933

[15] Okada Y Tanaka H Takeuchi H Yoshida O. Papillary adenocarcinoma in a seminal vesicle cyst associated with ipsilateral renal agenesis: a case report. J Urol. (1992) 148:1543–5. 10.1016/S0022-5347(17)36964-1 1433569

[16] Kim Y Baek HW Choi E Moon KC. Squamous cell carcinoma of the seminal vesicle from Zinner syndrome: a case report and review of literature. J Pathol Transl Med. (2015) 49:85–8. 10.4132/jptm.2014.10.28 25812665 PMC4357411

[17] Basillote JB Shanberg AM Woo D Perer E Rajpoot D Clayman RV. Laparoscopic excision of a seminal vesicle cyst in a child. J Urol. (2004) 171:369–71. 10.1097/01.ju.0000102300.07368.5c 14665933

[18] Moore CD Erhard MJ Dahm P. Robot-assisted excision of seminal vesicle cyst associated with ipsilateral renal agenesis. J Endourol. (2007) 21:776–9. 10.1089/end.2006.0279 17705770

